# Oral bioavailability and pharmacokinetic study of cetrizine HCl in Iranian healthy volunteers

**Published:** 2009

**Authors:** K. Derakhshandeh, M. Mohebbi

**Affiliations:** Department of Pharmaceutics, College of Pharmacy, Kermanshah University of Medical Sciences, Kermanshah, I.R. Iran

**Keywords:** Cetrizine, Pharmacokinetics, Bioequivalence, HPLC

## Abstract

The objective of the present study was to evaluate the pharmacokinetic parameters and bioavailability of a selective histamine (H1)-receptor antagonist, cetirizine hydrochloride (CTZ), following administration of a single oral dose of the drug. The properties of a test compound were compared with those of a reference product in a randomized cross-over study in 12 volunteers. Blood samples were collected at selected time intervals up to 24 h and plasma concentrations of CTZ were determined using a validated HPLC method. Pharmacokinetic parameters including T_1/2_, T_1/2_(abs), K, K_a_, T_max_, C_max_, V_d_/F, Cl/F, AUC_0-24_, AUC _0-∞_ and MRT were determined from plasma concentration-time profiles for tested products and found to be in good agreement with previous reports. The analysis of variance did not show any significant differences between the test and reference products. The confidence intervals for the ratio of C_max_ (95-110%), AUC_0-24_ (91-112%) and AUC_0-∞_ (92-109%) for the test and reference products were within the acceptable interval of 80-125%. ANOVA assessment of logarithmically transformed data did not reveal any significant subject, period or sequence effects. It was, therefore, concluded that the two products were bioequivalent and could be used interchangeably.

## INTRODUCTION

Cetirizine hydrochloride (CTZ), a human metabolite of the piperazine H1-receptor antagonist hydroxyzine, is used to treat seasonal allergic rhinitis, chronic idiopathic urticaria, perennial allergic rhinitis, allergic asthma, physical urticaria, and atopic dermatitis (1–3). The antihistaminic activity of CTZ, a secondgeneration antihistamine, has been demonstrated in a variety of animal and human models. *In vitro* receptor binding studies have shown no measurable affinity for other H1 receptors and negligible anticholinergic and antiserotonergic activities. Autoradiographic studies with radiolabeled cetirizine in rat have shown negligible penetration into the brain and the drug does not significantly occupy cerebral H1 receptors (1–5)

CTZ is rapidly absorbed from the gastrointestinal tract and peak plasma concentration is approximately 300 ng/ml one h after a 10 mg orally administered dose. The onset of activity occurs within 20 to 60 min and persists for at least 24 h following a single dose. The rapid onset of action and a once-daily dosing regimen are important advantages of this drug. Bioavailability is unchanged and time to peak plasma concentrations is delayed when administered with food (3–5).

CTZ is approximately 93% bound to plasma proteins, and has a plasma elimination half life of approximately 8-9 h which does not change with multiple dosing. Its Pharmacokinetic is dose-independent and plasma levels are proportional to the dose administered over the clinically studied range of 5 to 20 mg (3–5).

CTZ is less extensively metabolised than other antihistamines and approximately 60% of an administered dose is excreted unchanged in 24 h. Its high bioavailability associated with generally low inter-subject variations in blood concentration is attributable primarily to firstpass metabolism. Only one metabolite which is the product of oxidative dealkylation of the terminal carboxymethyl group with a negligible antihistaminic activity has been identified in human (3–5).

The primary aim of this study was to evaluate the pharmacokinetic parameters of CTZ after single oral administration of tablets in the present population. *In vitro* dissolution and *in vivo* bioavailability and pharmaco-kinetic profiles of a new generic formulation of this drug were compared with those of a reference product. A sensitive and repro-ducible HPLC analysis method for the determination of drug in human plasma was developed and used in the pharmacokinetic study.

## MATERIALS AND METHODS

### Drug preparation

Two commercial formulations including CTZ 10 mg tablets (Kimidaru, Iran) and Zyrtecset 10 mg tablets (UCB pharma S.A. France) were used as the test and reference products, respectively.

### In vitro study

Dissolution data were obtained on 12 tablets of each product using rotating paddles at 100 rpm (apparatus II). Dissolution medium was 900 ml of phosphate buffer (pH 2.5) which maintained at 37 ± 0.5 °C. Aliquots (5 ml) were taken for analysis at appropriate time intervals up to 45 min and diluted 1:10 with medium before analysis. Drug concentrations were measured spectrophotometrically at 229 nm. The similarity of two dissolution profiles was compared by Scale Up and Post Approval Changes (SUPAC) guidelines. The method was first reported by Moore and Flanner (6,7). The mean dissolution time (MDT) was calcul-ated using the following equation as applied previously to compressed tablet formulations(7):

Eq. 1 $$\mathrm{MDT}\;=\;\sum_{i}\;T_{i}\;{\mathrm{∆M}}_{i}/\sum_{i}\;{\mathrm{∆M}}_{i}$$

Where T_i_ is the midpoint of the time period during which the fraction Δ*M*_i_ of the drug is released from the dosage form(8)

### In vivo study

#### Subjects

Twelve healthy male volunteers with a mean age of 29 ± 2.3 years, mean weight of 75.3 ± 9.5 kg and mean height of 172.8 ± 7.6 cm, participated in the study after giving written informed consent about the nature and implications of the trial. All volunteers had normal examination and clinical laboratory test results, and none was on any medications for at least two weeks prior to and during the period of the study.

#### Drug administration and blood sampling

The study was conducted according to a double blind, randomized, cross-over design in which fasting subjects took a single oral dose of 10 mg (as one tablet) of either CTZ or the reference product with 250 ml of tap water in each period of the study. The washout period in this study was 1 week.

Blood samples (5 ml) were drawn just before and at 0.5, 1, 1.5, 2, 2.5, 3, 4, 6, 8, 10, 12 and 24 h after the drug administration and collected in heparinized tubes. Samples were centrifuged at 3000 rpm for 5 min and plasma stored at -20 °C until analysis.

### Cetrizine quantitation in human plasma

#### HPLC instrumentation

The chromatographic system consisted of the following components: two Shimadzu LC-10ADVP pumps, a Shimadzu DG14A degasser, a Shimadzu SPD-10AVP variable wavelength detector and a Shimadzu SCL-10AVP system controller. Chromatographic analyses were performed at 55°C; using a Shimadzu Shim-Pack C8 pre-column (10 mm × 4 mm i.d., 5 μm particle size) and a Shim-pack C18 analytical column (250 mm × 4 mm i.d., 5 μm particle size). The mobile phase consisted of methanol-water (60:40 v/v) (pH 3, adjusted by adding HCl 0.1 N) and was pumped at a flow rate of 1.2 ml/min. The analytes were detected at 229 nm.

#### Sample preparation

Sample preparation was performed by liquid-liquid extraction method using ethyl acetate. To 1 ml of human plasma, were added 50 μl of oxazepam solution as internal standard (10 μg/ml in metanol) and 3 ml of ethyl acetate. The mixture was vortex-mixed for 1 min and subsequently centrifuged for 10 min at 6000 rpm at ambient temperature. The supernatant layer was transferred into a glass tube and the procedure was repeated three times. The combined extracts was evaporated at 40°C under vaccume using a concentrator instrument (eppendorf, Germany), until a completely dried residue was left over. A volume of 50 μl of the mobile phase was added to the residue, and after vortex mixing for 30 s an aliquot of 20 μl of the solution was injected into the HPLC system.

### Validation of the assay

Validation was accomplished through determination of linearity, recovery, quantification limit, precision, accuracy, specificity and stability (9,10). A full validation (three analytical runs) for the analysis of CTZ in human plasma was completed. An eight point calibration curve (ranging from 10 to 500 ng/ml) was constructed by plotting the peak area ratio of analyte to the internal standard versus CTZ concentrations.

Specificity for an assay ensures that the signal measured comes from the substance of interest, and that there is no interference from excipient and/or degradation products and/or impurities.

The reproducibility of the analytical procedure was evaluated by determining the intraday and inter-day relative standard deviations (RSDs) at three concentrations using human plasma.

The absolute recoveries of CTZ were determined at concentrations of 10-500 ng/ml prepared in mobile phases in three different runs (Table 1). The limit of quantitation LOQ was defined as the lowest concentration at which the precision expressed by the RSD was lower than 15%. The accuracy which was expressed as the relative difference between the measured value and the actual value was also considered to be lower than 15%. The limit of detection (LOD) which is quoted as the concentration yielding a signal-to-noise ratio of 3:1 was confirmed by analyzing a number of samples near this value.

**Table 1:** Recovery efficiency of CTZ in extraction solvent during sample preparation process (n=5).

Nominal concentration (ng/ml)	Calculated concentration (ng/ml)	Recovery (%)
10	6.55 ± 0.15	65.59
50	34.9 ± 1.65	69.95
250	158 ± 10.2	63.30
500	326 ± 25.7	65.29

Mean ± S.D	-----	66.03 ± 2.8

**Table 2:** Intra-day assay precision and accuracy of the HPLC method for the determination of CTZ in human plasma*

Nominal concentration (ng/ml)	Calculated concentration (ng/ml)	Precision RSD (%)	Accuracy Error (%)
10	9.95	7.36	-0.50
50	51.2	7.02	2.38
250	243	8.36	-2.64
500	512	6.15	2.31

* Samples were analyzed in five replicates for each concentration per day

The chemical stability of CTZ in biomatrix at three different concentrations of 50, 100 and 500 ng/ml was determined following three consecutive freeze-thaw cycles, in which the frozen plasma samples were kept at room temprature for 30 min before subsequent freezing.

### Pharmacokinetic analysis

CTZ pharmacokinetic parameters were determined by noncompartmental methods. The elimination rate constant (K) was estimated by the least-square regression of plasma concentration-time data points lying in the terminal log-linear region of the curves. The elimination half life was calculated as 0.693 divided by K. The area under the plasma concentration-time curve from time zero to the last measurable concentration at time t (AUC_0-t_) was calculated using the trapezoidal rule. The area was extrapolated to infinity (AUC0-∞) by addition of C_t_/K to AUC_0-t_ where C_t_ is the last measured drug concentration. Peak plasma concentration (C_max_) and time to peak concen-tration (T_max_) were determined by inspection of the individual subject concentration time curves. The relative bioavailability of the test formulation was estimated as the AUC_0-∞_ratio of the test to the reference product. The Wagner-Nelson method was used to estimate fractional absorption (11). Fractional absorbed data for each subject and treatment was used for estimation of the apparent absorption rate constant (K_a_). Absorption half-life (T_1/2_ _(abs)_) was calculated by 0.693/K_a_.

Apparent oral clearance (Cl/F) and apparent volume of distribution (V_d_/F) were calculated by Eq. 2 and 3, respectively:

Eq. 2 $$C1/F\;=\;\mathrm{Dose}/{\mathrm{AUC}}_{0-\infty }$$

Eq. 3 $$V_{d}/F\;=\;C1/K$$

Mean residence time (MRT), the average time for all the drug molecules to reside in the body was estimated according to the following equation (12):

Eq. 4 $$\mathrm{MRT}=\;{\mathrm{AUMC}}_{0-\infty }/{\mathrm{AUC}}_{0-\infty }$$

Where AUMC is the area under the first moment of plasma drug concentration.

### Statistical analysis

The *in vitro* dissolution data was compared by two-tailed student’s t-test. Logarithmic transformation of AUC_0-24_, AUC_0-∞_, and C_max_ were compared by the analysis of variance (ANOVA) for a crossover design followed by 90% confidence interval test for the arithmetic mean pharmacokinetic parameters of CTZ formulations. The non-parametric ‘wilcoxon test’ was also performed for analyzing untransformed T_max_data.

## RESULTS

### In vitro study

**Fig. 1:**
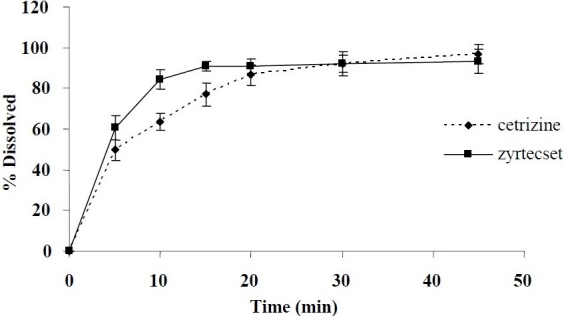
*In vitro* release profile of CTZ products in phosphate buffer (pH 2.5), data represent means (n=12).

The MDTs of the test and reference products were 8.39 ± 1.82 and 5.31 ± 1.59 min, respectively (Fig. 1). Statistical analysis showed significant differences between the MDT for two dosage forms (*P*<0.05). More comparison on dissolution profiles of the two products was done by SUPAC test. The calculated values of F_1_(dissimilarity factor) and F_2_(similarity factor) were 17.85 and 43.09, respectively. In spite of a significant difference between the MDT and F values of the two products, the drug content of both formulations was largely released within 20 min.

Since more than 80% of the drug was released before 45 min, the release characteristics of the two brands could be considered pharmaceutically equivalent and met the general single-point dissolution USP requirements.

### Development of the analytical method for cetrizine quantitation in plasma

**Fig. 2:**
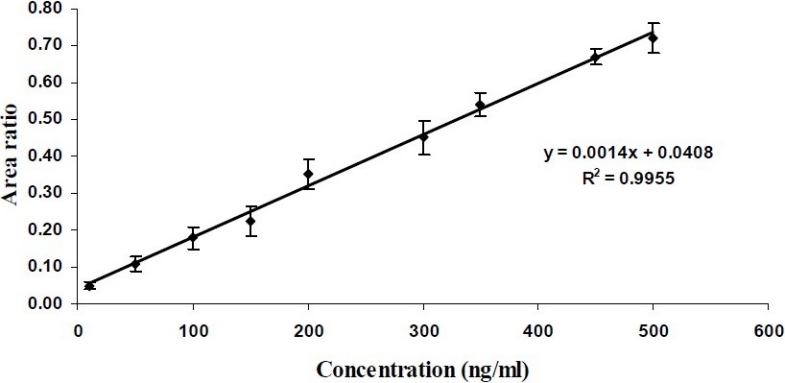
Linearity of the standard calibration curve of CTZ in human plasma samples (n=3).

**Fig. 3:**
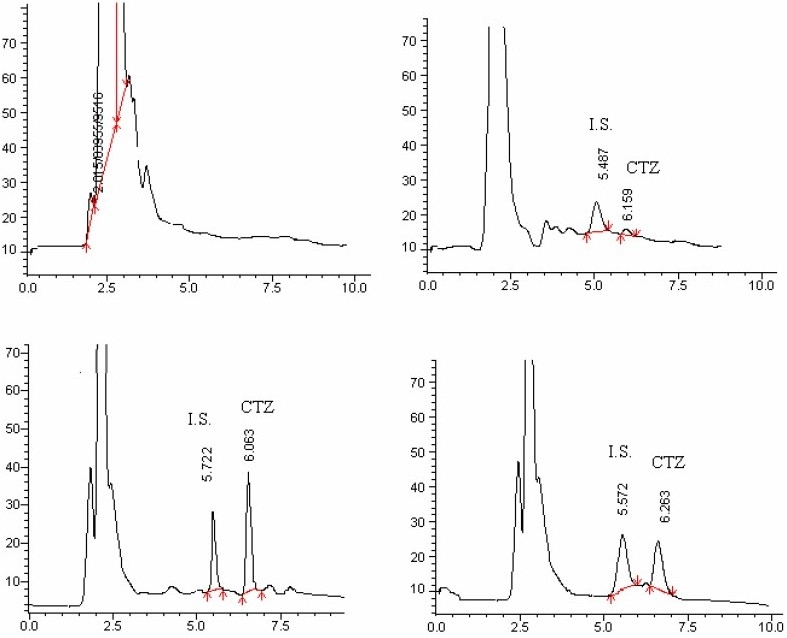
Representative HPLC chromatograms for CTZ and I.S. in human plasma samples: (A) blank plasma obtained from healthy volunteers; (B) blank plasma spiked with CTZ (10 ng/ml) and I.S. (10 μg/ml); (C) blank plasma spiked with CTZ (250 ng/ml) and I.S. (10 μg/ml); and (D) plasma from healthy volunteer, 5 h after an oral administration of a single dose tablet containing 10 mg CTZ.

The proposed method was considered suitable for CTZ quantification in plasma samples. The method showed good linearity in the range of 10 to 500 ng/ml of drug concentrations [Y = 0.0014 (± 0.0002)X - 0.0408 (± 0.0071), r^2^= 0.9955] (Fig. 2). As it is shown in Fig. 3, chromatograms with good resolution and free of endogenous or interfering peaks were obtained. The recovery, limit of quantification and detection limit, were 66.60 ± 2.65%, 10 ng/ml and 2.5 ng/ml, respectively. The Intra-day and inter-day variations were between 6.15 and 8.36% and between 7.51 and 10.16%, respectively (Table 1–3). Plasma samples were stable at 4°C and -20°C for 90 days and reconstituted samples were stable for 24 h at room temperature. Drug in plasma samples was also stable following freeze-thaw cycles.

**Table 3:** Inter-day assay precision and accuracy of the HPLC method for the determination of CTZ in human plasma*

Nominal concentration (ng/ml)	Calculated concentration (ng/ml)	Precision RSD (%)	Accuracy Error (%)
10	9.940	7.51	-0.60
50	51.81	10.2	3.62
250	259.3	8.37	3.72
500	518.6	6.15	3.73

* Validation study, n=5 days, six replicates per day

### Bioequivalence evaluation

**Fig. 4:**
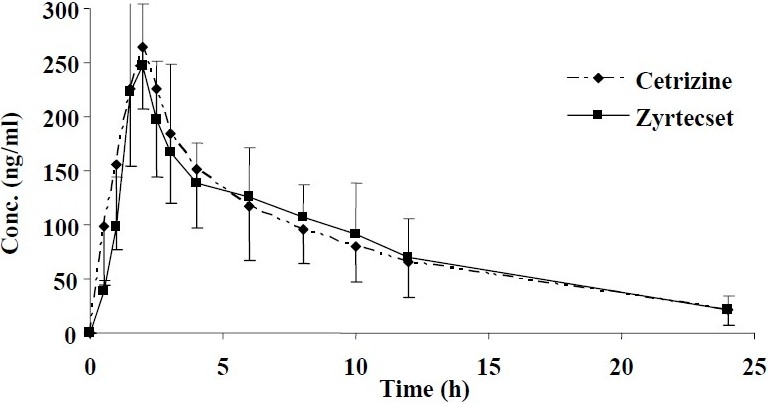
Average plasma concentrations of CTZ after oral administration of a 10 mg tablet of either the reference (Zyrtecset® UCB pharma) or test (Cetrizine, Kimidaru) product to 12 healthy volunteers. (Each point represents mean ± SD).

Average plasma concentration of drug versus time course after oral administration of the reference (Zyrtecset) and test (cetrizine) products in 12 healthy volunteers are shown in Fig. 4.

Pharmacokinetic parameters calculated from individual plasma level-time data are shown in Table 4.

**Table 4:** Mean Pharmacokinetic parameters (± SD) of CTZ following administration of a single dose CTZ (10 mg) in two different oral formulations (n=12).

Parametrs	Test product	Reference product	t-test
C_max_ (ng/ml)	273 ± 24.5	266 ± 24.8	NS
T_max_ (h)	1.96 ± 0.25	1.75 ± 0.26	NS
AUC_0-12_ (ng.h/ml)	2024 ± 247	2006 ± 275	NS
AUC_0-∞_ (ng.h/ml)	2534 ± 378	2526 ± 360	NS
T_1/2_ (h)	7.57 ± 1.76	7.15 ± 1.33	NS
T_1/2_ (abs) (h)	0.49 ±0.15	0.77 ±0.29	*P*<0.05
K (h^-1^)	0.10 ± 0.02	0.10 ± 0.03	NS
K_a_ (h^-1^)	1.61 ± 0.62	0.99 ± 0.29	*P*<0.05
Cl/F (l/Kg/h)	0.06 ± 0.01	0.06 ± 0.01	NS
V_d_/F (l/Kg)	0.68 ± 0.12	0.66 ± 0.13	NS
MRT	9.47 ± 1.52	9.47 ± 0.92	NS

NS = Non Significant (*P*>0.05)

In order to confirm the bioequivalency of the two products, the 90% confidence intervals for arithmetic mean of test/reference, indivi-dual ratios of C_max_, AUC_0-24_, AUC_0-∞_, and Cmax/AUC_0-∞_ were calculated. All values were found to be within the conventional bioequi-valence ranges of 0.8-1.2 (Table 5 and 6). Wilcoxon Signed Rank non-parametric analysis did not reveal significant differences between T_max_ values (*P* = 0.137). As shown in Table 6, the parametric point estimate of the difference (T-R) for T_max_ is 0.21 h and thus within the stipulated bioequivalence range of ± 0.35 h (± 20% of the mean of the reference product). No significant differences were observed between the C_max_, T_max_, AUC_0-24_, AUC_0-∞_, T_1/2_, K, Cl/F, V_d_/F and MRT of the two products (*P*>0.05), however we observed some differences between K_a_and T_1/2α_ (Table 4).

**Table 5:** ANOVA for assessment of the drugs, subjects and period effects, and 90% CI for the test/reference ratio of C max, AUC (0-t) and AUC (0-∞), using logarithmic transformed data, after administration of a single dose CTZ (10 mg) in two different oral formulations (n=12).

Parametrs	Variation source (*p-value*)			90%CI (%)
C_max_	0.527	0.408	0.701	95-110
AUC_0-t_	0.872	0.353	0.520	91-112
AUC_0-∞_	0.941	0.317	0.866	92-109

**Table 6:** Parametric 90% confidence intervals for the mean pharmacokinetic parameters of CTZ formulations.

Parametrs	Test / Reference	
	means	90%CI
C_max_	1.03	0.97-1.10
AUC_0-t_	1.03	0.94-1.12
AUC_0-∞_	1.01	0.93-1.09
C_max_/AUC_0-∞_	1.05	0.94-1.16
T_max_ difference	0.21	P_value_ = 0.137*

* Wilcoxon nonparametric test

## DISCUSSION

The bioequivalence and pharmacokinetics of two CTZ products were studied in 12 volunteers following a single dose of 10 mg tablets. The plasma concentration of CTZ was determined using a simple, sensitive and reproducible HPLC method, developed in this laboratory. Increased sensitivity, evident from lower LOD and LOQ, and the high recovery of extraction of the HPLC assay are comparable to the published methods (13–15).

*In vitro* CTZ release profiles of the two products were also characterized in the present study. Although the two products released their drug content differently at early stage of dissolution, the content of both reference and test products was almost entirely released within 20 min. This may translate into similar release pattern *in vivo*.

The mean plasma concentration-time profiles of CTZ following oral administration of both products are shown in Fig. 4. The blood sampling schedule was designed according to FDA regulations (16). Sampling was accomplished up to at least 3 terminal elimination half lives of the drug in the present study, and the time intervals between sampling did not exceed one terminal half life of CTZ. The AUC_0-24_ was greater than 80% of the AUC_0-∞_ in all subjects, indicating adequate sampling time and intervals to estimate the extent of absorption. The power of ANOVA was estimated to be >0.8 at 90% CI, indicating that 12 subjects would suffice for the purposes of the study.

Two-way ANOVA for cross-over design was performed on log-transformed data to assess the effect of formulations, periods, sequences and subjects nested in sequence on the parameters. The effect of periods, sequence or treatment did not differ for any of pharmacokinetic parameters.

The results in this study showed that CTZ pharmacokinetic followed one compartment kinetics with a rapid absorption rate (0.49, 0.77 h) and elimination phase (7.57, 7.15 h). The average plasma decay curves of two formulations were similar and pharma-cokinetic parameters of T_max_, C_max_, T_1/2_ didn’t show any significant differences (Table 2). Maximum plasma concentrations of CTZ were about 266 and 273 ng/ml and AUC_0-∞_ values were 2526 and 2534 ng.h/ml for the reference and test products, respectively.

In a study conducted by Lefebvre (17) and coworkers the pharmacokinetics of CTZ in elderly and young healthy volunteers after a single 10 mg oral intake was investigated. In young healthy volunteers, about 60% of drug was excreted in the urine unchanged. Mean plasma concentrations were slightly higher in elderly subjects. C_max_(362 ng/ml), T_max_(1.30 h), terminal half-life (11.8 h) and AUC_0-∞_, (4316 ng.h/ml) in the elderly subjects were somewhat higher than those of the young subjects (C_max_: 337 ng/ml, T_max_: 1.12 h, terminal half life: 10.6 h, AUC_0-∞_: 3721 ng.h/ml). The mean cumulative urinary excretion at 32 h was significantly lower in the elderly subjects. The slight differences in pharmacokinetics of CTZ between young and elderly subjects after single oral intake could be attributed to the decreased renal clearance in the elderly (17). The elimination half-life of CTZ was prolonged in patients with renal and chronic liver insufficiency, compared with age-matched individuals with normal renal function (19.0-20.9 vs. 7.4 h, respectively).(18,19).

The pharmacokinetic parameters were in agreement with those reported by some other researchers.

Pharmacokinetic parameters of two CTZ formulations were evaluated in healthy male volunteers. The values of C_max_, T_max_, t_1/2_, AUC_(0-t)_, AUC_(0-∞)_ for test and reference products were estimated to be 301.0 ± 36.6 and 285.4 ± 39.1 ng/ml, 1.0 ± 0.3 and 1.1 ± 0.5 h, 8.06 ± 1.3 and 7.82 ± 1.3 h, 2115.9 ± 523.5 and 2228.89 ± 740.3 ng.h/ml, 2400.8 ± 666.6 and 2368.6 ± 492.9 ng.h/ml, respectively (20). In another study the values obtained for C_max_, T_max_, t_1/2_and AUC_(0-s)_, for CTZ were 381 ± 73.7 ng/ml, 0.75 (0.5-3.0) h, 7.82 ± 1.43 h and 3472 ± 719 μg.h/ml, respectively (21). These data are also in accordance with those estimated in our report.

No major adverse effects were observed during the study period. Only minor sleepiness and tiredness was observed 6-8 h after drug administration. Therefore, the results are indicative of a good safety profile of the regimen. However, this study is limited in that only a small number of safety parameters were evaluated in this group. This effect must be taken into account in future pharmacodynamic studies in this age group.

## CONCLUSION

In this study, the *in vitro* dissolution and *in vivo* pharmacokinetics of two formulations of CTZ, following administration of a single oral dose, were investigated. Plasma concentration profiles were characterized in 12 healthy volunteers and the pharmacokinetic parameters of CTZ following a single oral dose administration were estimated. An optimized HPLC method, which provides a sensitive and accu-rate means for determination of CTZ in micro volume of human plasma, was also developed. The 90% confidence intervals for the ratio of C_max_, AUC_0-t_ and AUC_0-∞_values for the test and reference products were within the 80-125% interval proposed by FDA. It was concluded that the two CTZ products were bioequivalent in their rate and extent of absorbtion.
